# Comparative efficacy of image-guided techniques in cardiac resynchronization therapy: a meta-analysis

**DOI:** 10.1186/s12872-021-02061-y

**Published:** 2021-05-24

**Authors:** Xiao Hu, Hai Xu, Shameer Raaj Avishkar Hassea, Zhiyong Qian, Yao Wang, Xinwei Zhang, Xiaofeng Hou, Jiangang Zou

**Affiliations:** 1 Department of Cardiology, The First Affiliated Hospital of Nanjing Medical University, No.300, Guangzhou Road, Nanjing, 210029 China; 2 Department of Cardiology, The Affiliated Huaian No.1 People’s Hospital of Nanjing Medical University, Huaian, Jiangsu China; 3 Department of Cardiology, Staten Island University Hospital / Northwell Health, 475 Seaview Drive, Staten Island, NY 10305 USA

**Keywords:** Cardiac resynchronization therapy, Image-guided, CRT response, Heart failure

## Abstract

**Background:**

Several studies have illustrated the use of echocardiography, magnetic resonance imaging, and nuclear imaging to optimize left ventricular (LV) lead placement to enhance the response of cardiac resynchronization therapy (CRT) in heart failure patients. We aimed to conduct a meta-analysis to determine the incremental efficacy of image-guided CRT over standard CRT.

**Methods:**

We searched PubMed, Cochrane library, and EMBASE to identify relevant studies. The outcome measures of cardiac function and clinical outcomes were CRT response, concordance of the LV lead to the latest sites of contraction (concordance of LV), heart failure (HF) hospitalization, mortality rates, changes of left ventricular ejection fraction (LVEF), and left ventricular end-systolic volume (LVESV).

**Results:**

The study population comprised 1075 patients from eight studies. 544 patients underwent image-guided CRT implantation and 531 underwent routine implantation without imaging guidance. The image-guided group had a significantly higher CRT response and more on-target LV lead placement than the control group (RR, 1.33 [95% CI, 1.21 to 1.47]; *p* < 0.01 and RR, 1.39 [95% CI, 1.01 to 1.92]; *p* < 0.05, respectively). The reduction of LVESV in the image-guided group was significantly greater than that in the control group (weighted mean difference, − 12.46 [95% CI, − 18.89 to − 6.03]; *p* < 0.01). The improvement in LVEF was significantly higher in the image-guided group (weighted mean difference, 3.25 [95% CI, 1.80 to 4.70]; *p* < 0.01). Pooled data demonstrated no significant difference in HF hospitalization and mortality rates between two groups (RR, 0.89 [95% CI, 0.16 to 5.08]; *p* = 0.90, RR, 0.69 [95% CI, 0.37 to 1.29]; *p* = 0.24, respectively).

**Conclusions:**

This meta-analysis indicates that image-guided CRT is correlated with improved CRT volumetric response and cardiac function in heart failure patients but not with lower hospitalization or mortality rate.

**Supplementary Information:**

The online version contains supplementary material available at 10.1186/s12872-021-02061-y.

## Background

Heart failure (HF) affects an approximate 37.7 million people worldwide [1]. Although drug therapy for HF has made significant progress in recent years [2, 3], most patients continue to suffer from poor prognosis and high fatality rate [4]. Cardiac resynchronization therapy (CRT) are now being widely accepted as a significant component of standard HF therapy. In most patients with appropriate indications, CRT reduces clinical symptoms, improves exercise tolerance, and reverses cardiac remodeling [5]. However, a substantial number of patients have a poor response to CRT [6]. Several studies have shown that the area of left ventricular (LV) with the most delayed mechanical activation is the ideal site for LV lead placement [7, 8]. Therefore, the target vessel position of the LV lead is an essential factor in determining CRT response. It remains technically challenging to locate the LV lead in this ideal area through coronary venography. Several image-guided methods have been proposed to locate this area, including echocardiography (ECHO) [9, 10], and cardiac magnetic resonance imaging (CMR) [11]. Speckle tracking ECHO (STE) provides myocardial strain measurement to distinguish zones of scarred myocardium, as well as vital features of dyssynchrony [12]. Phase analysis (PA) technique based on the single-photon emission computed tomography myocardial perfusion imaging (SPECT MPI) is another newly innovative imaging modality, with potential to identify LV mechanical dyssynchrony, latest-excited sites, and myocardial scar load [13]. The 13-segmentation polar map based on this PA technique is capable of displaying a mean phase angle, and thus allowing the identification of systolic dyssynchrony as well as the late contracting segments. To date, several studies, despite inconsistency in research strategies and reporting mechanisms, have reported that image-guided techniques were associated with improved CRT efficacy. The primary objective of this study was to evaluate the evidence surrounding this proposed efficacy improvement secondary to imaging guided CRT placement. To that end, we undertook a meta-analysis of the published literature pertaining to the documentation of clinical outcomes from image-guided CRT implantation in HF patients.

## Methods

### Eligibility and search strategy

A comprehensive literature search of the PubMed, Cochrane library, and EMBASE databases (from inception to November 2020) were conducted to identify primary studies reporting associations between image-guided LV lead placement and CRT efficacy. Keywords used for literature search included: “left ventricular lead placement”, “cardiac resynchronization therapy”, “image-guided”, “echocardiography-guided”, “multimodality imaging”, and “SPECT-guided”. Additionally, pertinent publications found by review of citation lists of identified publication were examined. The meta-analysis was subsequently performed in adherence to the Preferred Reporting Items for Systematic Reviews and Meta-Analyses (PRISMA) statement (Additional file 1: Table S1)[14].

### Inclusion and exclusion criteria

Publications identified from abovementioned databases were individually screened by titles, abstracts, methods and results to meet the following inclusion criteria: (1) prospective, randomized controlled trials (RCTs) or observational (prospective or retrospective cohort) studies; (2) patients recruited with confirmed HF diagnoses; (3) patients received a CRT pacemaker (CRT-P) or a CRT defibrillator (CRT-D) device; (4) association between image-guided LV lead placement and CRT response reported; (5) CRT response used as a measure of outcome; (6) the period of follow-up was ≥ 6 months. The exclusion criteria applied were: (1) RCTs without treatment groups; (2) patients were treated with other interventions.

### Quality assessment

The quality for the included RCTs was assessed based on the Cochrane Risk of Bias tool by Review Manager 5.1. Quality item was classified as high risk, low risk, or unclear risk. The assessment is divided into six parts (key points of quality assessment are listed in Table 1). The studies were regarded as high quality, low quality, or moderate quality with the following principles: (1) if both randomization and allocation concealment were evaluated as a low risk of bias, studies were regarded as high quality; (2) if either allocation concealment or randomization was evaluated as a high risk of bias, studies were regarded as low quality; (3) remaining studies were regarded as moderate quality. The quality of the included observational studies was evaluated using the Newcastle–Ottawa Scale based on the methods of selection (4 stars), comparability (2 stars), and outcome (3 stars).

**Table 1:** The methodological quality of RCTs based on the Cochrane handbook

Study	A	B	C	D	E	F
Saba	+	?	+	−	+	?
Khan	+	+	+	−	+	+
Sommer	+	−	+	+	−	+
Zou	+	−	+	−	+	+

A, randomization sequence generation; B, allocation concealment; C, blinding of participants, personnel and outcome assessment; D, Incomplete outcome data; E, selective reporting; F, other bias; +, yes; −, no; ?, unclear

### Data extraction

The following pre-determined findings were identified and recorded when provided from each of the eligible publications: (1) general information: publication year, lead author(s), and the origin of the population; (2) study characteristics: subject age and gender, numbers of cases, mean follow-up duration, study design, QRS duration, New York Heart Association (NYHA) grades, and types of intervention performed; (3) assessment of cardiac functions and clinical outcomes: mean and standard deviation (SD) of Left ventricular ejection fraction (LVEF) and Left ventricular end-systolic volume (LVESV), concordance of LV lead to the latest sites of contraction (concordance of LV), CRT response, HF-related hospitalization, and all-cause mortality. The data extraction was performed by two investigators independently. A consensus was reached for any disagreement based on discussions between two researchers or the involvement of a third independent investigator. The CRT response was defined as LV reverse remodeling (≥ 15% reduction in LVESV) at 6 months.

### Statistical analysis

Statistical analysis was performed using Review Manager 5.3.5 (The Cochrane Collaboration, Oxford, England). The relative risk ratio (RR) and its 95% confidence interval (CI) were used to compare CRT response, concordance of LV, mortality and HF hospitalization rates between the image-guided CRT and the standard CRT groups. The weighted mean difference (WMD) and its 95% CI were calculated to assess the differences in LVESV and LVEF between both groups. The heterogeneity among the analyzed studies was tested by the Cochrane Q statistic and the I^2^ value. To investigate additional factors impacting these results, we conducted a subgroup analysis based on country, study design, as well as LVEF and LVESV ranges. P values were calculated between subgroups by the interaction test. To evaluate the robustness of pooled results, we performed sensitivity analysis by excluding low or specific studies. The fixed-effect model was used when no statistical heterogeneity between studies (P > 0.1, I^2^ < 50%) was detected and the random-effects model was used when heterogeneity was deemed significant. Publication bias was visually assessed using funnel plots. Statistical significance was defined as P < 0.05.

## Results

### Search Results

**Fig. 1:**
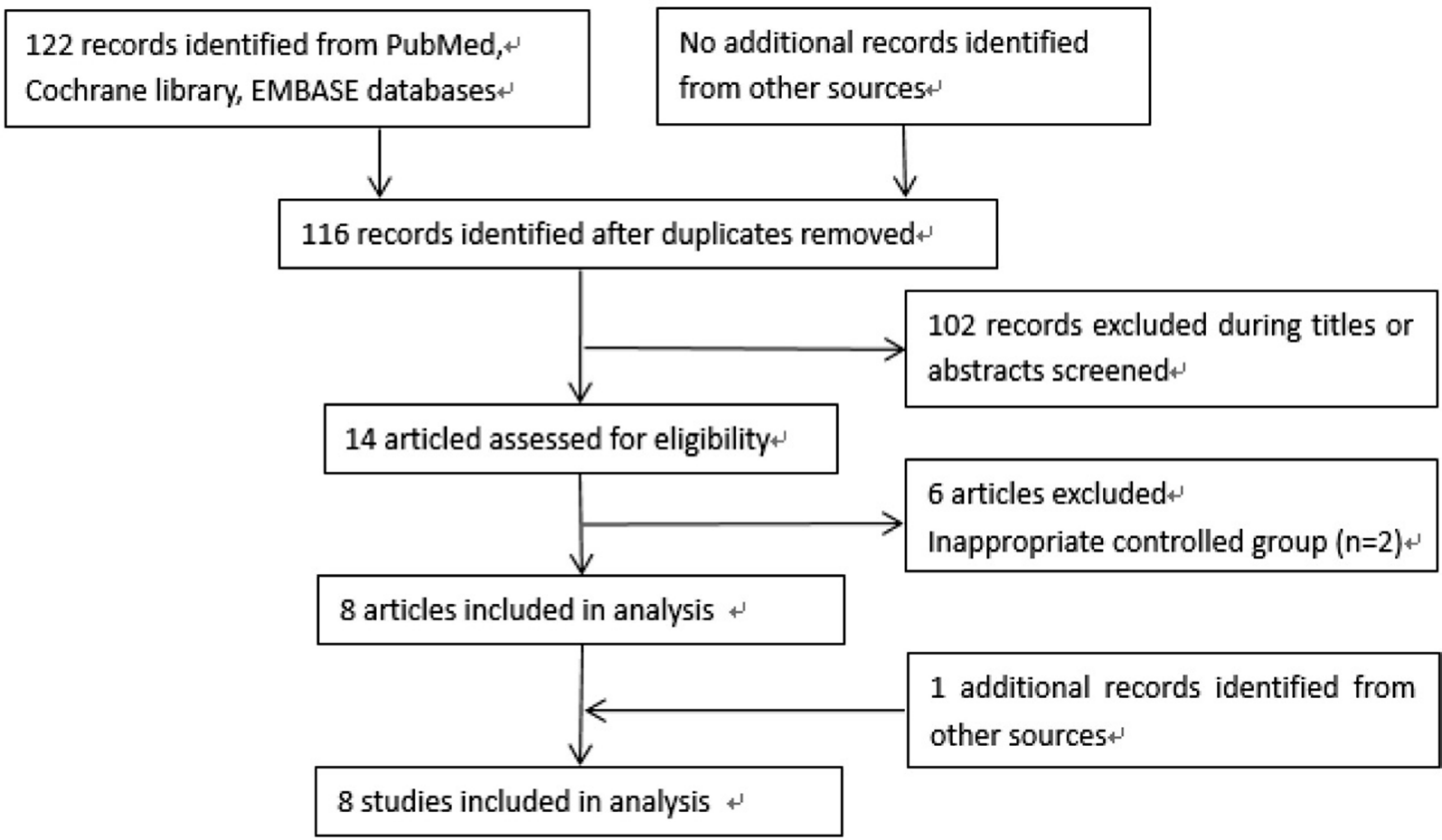
Flow diagram for study selection

The search strategy generated 122 publications, of which 102 were excluded based on abstract review and titles. Two studies with inappropriately controlled groups were then excluded. Finally, eight studies (4 RCTs and 4 observational studies) were included [15–22]. The study population encompassed by the 8 publications identified as described above consisted of 1075 patients. Flow diagram for study selection is presented in Fig. 1.

### Study characteristics

Baseline characteristics of included studies are shown in Table 2. The trials by Saba et al. [17] and Khan et al. [16] utlized STE, while Bai et al. [15] applied intracardiac ECHO coupled with vector velocity imaging (VVI). Bertini et al. [18] evaluated the role of CMR in CRT implantation. Sommer et al. [19] used multimodality imaging-guided (cardiac computed tomography, STE, and SPECT) techniques to guide LV lead placement to the ideal coronary sinus branch. In contrast, Salden et al. [20] assessed Real-time image-guided LV lead placement by fusion of fluoroscopy images with CMR images. The GUIDECRT trial by Zou et al. [21] validated the improvement of CRT efficacy guided by SPECT. Mele et al. [22] investigated the feasibility of LV lead placement directed by parametric two-dimensional STE with polar plots of the amplitude and timing of LV longitudinal strain. In total, 544 patients underwent image-guided CRT implantation and 531 underwent routine implantation without imaging guidance. Follow-up durations ranged from 6 months to 2 years. The outcome results of the individual study are shown in Table 3.

**Table 2:** Baseline characteristics of the included studies and patients

Study	Country	Study design	CRT intervention	Sample size	Age (year)	Gender male (%)	NYHA class	QRS duration (ms)	Definition of response
Bai et al. [15]	United States	Prospective study	Image-guided	50	66 ± 11	60	III or IV: 3.10 ± 0.30	153 ± 23	At least 1 class decrease of NYHA, increase > 20% in LVEF, reduction ≥ 15% in LVESV. fulfill at least 2 of the above 3 criteria
			Standard	54	64 ± 9	74	III or IV: 3.07 ± 0.26	155 ± 29	
Khan et al. [16]	United Kingdom	RCT	Image-guided	110	72 (65–76)	77	III/IV:95/15	157 (148–170)	A reduction ≥ 15% in LVESV
			Standard	110	72 (64–80)	80	III/IV:93/17	159 (146–170)	
Saba et al. [17]	United States	RCT	Image-guided	110	66 ± 11	70	II/III/IV:16/64/20	157 ± 27	A reduction ≥ 15% in LVESV or ≥ 5% increase in LVEF with no primary end point (death or first HF hospitalization)
			Standard	77	67 ± 13	78	II/III/IV:8/71/21	162 ± 27	
Sommer et al. [19]	Denmark	RCT	Image-guided	89	71 ± 9	78	II/III/IV:44/44/1	167 ± 22	Improvement in NYHA class, ≥ 10% increase in 6MWT distance, with no death or HF hospitalization
			Standard	93	71 ± 9	80	II/III/IV:40/48/5	165 ± 22	
Mele et al. [22]	Italy	Retrospective study	Image-guided	64	68.4 ± 9.0	77	II/III/IV:47/15/2	153.4 ± 23.6	A reduction > 15% in LVESV
			Standard	64	68.4 ± 11.1	86	II/III/IV:37/23/4	155.3 ± 16.1	
Bertini et al. [18]	Italy	Prospective study	Image-guided	50	67.3 ± 9.7	74	II/III/IV:29/17/4	156 ± 24	A reduction ≥ 15% in LVESV
			Standard	50	65.6 ± 8.4	76	II/III/IV:25/24/1	154 ± 30	
Salden et al. [20]	Netherlands	Prospective study	Image-guided	6	67 ± 3	50	II/III:5/1	165 ± 26	A reduction > 15% in LVESV
			Standard	9	69 ± 9	78	II/III:7/2	160 ± 22	
Zou et al. [21]	China	RCT	Image-guided	87	62.5 ± 11.5	68	II/III/IV:18/52/17	163.57 ± 23.63	A reduction > 15% in LVESV
			Standard	90	62.7 ± 11.2	72	II/III/IV:18/55/17	161.17 ± 24.16	

CRT, cardiac resynchronization therapy; LVESV, left ventricular end-systolic volume; LV, left ventricular; EF, ejection fraction; RCT, randomized controlled trial; NYHA, New York Heart Association

**Table 3:** Outcomes of the included studies

Study	CRT treatment	Concordance with the site of latest activation (%)	CRT response (%)	LVEF(%), mean ± SD			LVESV(ml), mean ± SD			Death (%)	HF hospitalization (%)
				Baseline	Follow up	Change	Baseline	Follow up	Change		
Bai et al. [15]	Image-guided	NR	41 (82)	23 ± 7	34 ± 10	11 ± 8.89^b^	172 ± 65	129 ± 65	− 43 ± 65	NR	NR
	Standard	NR	34 (63)	26 ± 6	32 ± 9	6 ± 7.94^b^	159 ± 74	141 ± 82	− 18 ± 78	NR	NR
Khan et al. 2012	Image-guided	Concordant: 63	72 (70)	23 ± 6	31 ± 9	8 ± 7	157 ± 56	111 ± 43	− 46 ± 33	NR	NR
		Adjacent: 26									
		Remote: 10									
	Standard	Concordant: 47	57 (55)	23 ± 7	28 ± 10	5 ± 8	154 ± 52	128 ± 50	− 26 ± 23	NR	NR
		Adjacent: 29									
		Remote: 25									
Saba et al. [17]	Image-guided	Concordant: 30	50 (57)	26 ± 6	38 ± 12.8^a^	12 ± 11	140 ± 59	110 ± 31^a^	− 30 ± 29	15 (13.6)	16 (14.5)
		Adjacent: 55									
		Remote: 15									
	Standard	Concordant: 12	22 (35)	26 ± 7	35 ± 11.45^a^	9 ± 10	144 ± 63	125 ± 52^a^	− 20 ± 25	15 (19.5)	21 (27.3)
		Adjacent: 54									
		Remote: 33									
Sommer et al. [19]	Image-guided	Concordant: 49	66 (74)	25 ± 6	37 ± 10.35^a^	12 ± 9	190 ± 70	156 ± 67^a^	− 34 ± 23	1 (1.1)	3 (3.4)
		Adjacent: 50									
		Remote: 1									
	Standard	Concordant: 43	54 (58)	24 ± 6	36 ± 9.08^a^	12 ± 8	198 ± 69	165 ± 56^a^	− 33 ± 23	2 (2.2)	1 (1.1)
		Adjacent: 54									
		Remote: 2									
Mele et al. [22]	Image-guided	NR	48 (64)	29.1 ± 5.9	39.0 ± 9.9	9.9 ± 8.6^b^	138.4 ± 41.8	107.5 ± 43.8	− 30.9 ± 38.9^b^	NR	NR
	Standard	NR	31 (64)	29.8 ± 5.0	35.3 ± 6.7	5.5 ± 6.0^b^	140.5 ± 43.1	124.4 ± 45.4	− 16.1 ± 44.3^b^	NR	NR
Bertini et al. [18]	Image-guided	Concordant: 58	39 (78)	29 ± 6	42 ± 11	13 ± 8.72^b^	142 ± 47	102 ± 45	− 40 ± 46.03^b^	NR	NR
		Adjacent: 36									
		Remote: 6									
	Standard	NR	28 (56)	29 ± 6	37 ± 9	8 ± 9.54^b^	148 ± 51	121 ± 55	− 27 ± 53.11^b^	NR	NR
Salden et al. [20]	Image-guided	Concordant: 50	6 (100)	27 ± 6	42 ± 6^a^	15 ± 5	175 (142–216)	NR	− 30 ± 10	NR	NR
		Adjacent: 50									
		Remote: 0									
	Standard	NR	NR	25 ± 5	35 ± 13.69^a^	10 ± 12	128 (96–169)	NR	− 19 ± 19	NR	NR
Zou et al. [21]	Image-guided	Concordant and adjacent: 85.5	66 (76)	26.69 ± 6.22	NR	NR	187.27 ± 77.94	139.07 ± 60.6^a^	− 48.2 ± 61.6	NR	NR
		Remote: NR									
	Standard	Concordant and adjacent: 62.4	57 (63)	27.28 ± 6.17	NR	NR	189.52 ± 76.83	160.62 ± 67.8^a^	− 28.9 ± 54.6	NR	NR
		Remote: NR									

NR, not reported; ml, milliliters; SD, standard deviation; HF, heart failure; other abbreviations as in Table 2

^a^Data were estimated based on the results of baseline and change from baseline

^b^Data were estimated based on the results of baseline and follow-up

### Quality assessment

One RCT reported the generation process of adequate random sequence and the allocation concealment [16]; therefore, it was regarded as high quality. Another three RCT studies were deemed moderate quality [17, 19, 21]. Based on the Newcastle–Ottawa Scale for risk-stratifying observational study biases, three studies received eight stars [15, 18, 20], and one study received seven stars [22]. Quality assessments of the included RCTs are presented in Table 1.

### Meta-analysis

#### CRT response

**Fig. 2:**
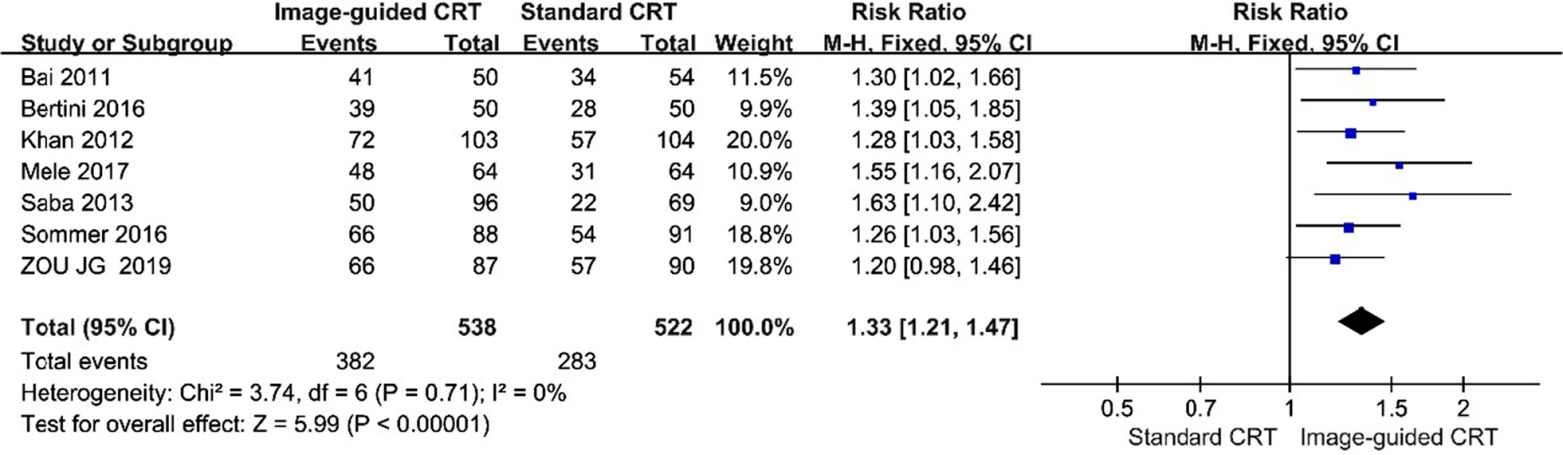
Forest plot of CRT response between groups. A fixed-effects model and Mantel–Haenszel method were used to pool data. Abbreviations: CI, confidence interval; CRT, Cardiac resynchronization therapy

Seven studies reported direct comparison of responses between image-guided CRT and standard CRT placements [15–19, 21, 22]. All seven studies showed that participants undergoing image-guided CRT had significantly higher CRT response rates. There was statistically a significant association between image-guided CRT placement and improved CRT responses (RR, 1.33 [95% CI, 1.21 to 1.47]; *p* < 0.01, Fig. 2), when compared to standard CRT treatment with null heterogeneity (P = 0.71; I^2^ = 0).

#### Improvement in LVEF

**Fig. 3:**
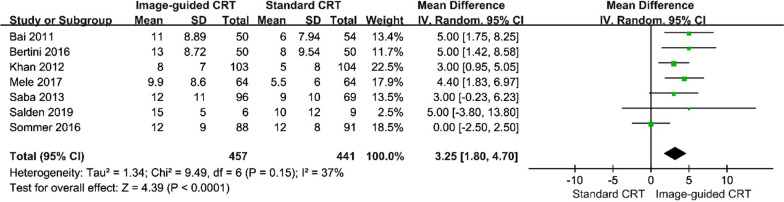
Forest plot of change in LVEF between groups. A random-effects model and inverse variance (IV) method were used to pool data. Abbreviations: SD, standard deviation; other abbreviations as in Fig. 2

Seven studies with available LVEF data were included for this meta-analysis [15–20, 22]. Among them, five studies demonstrated significant increases in LVEF in the image-guided CRT treatment group when compared to that in the standard CRT group [15, 16, 18, 20, 22]. In a pooled analysis of all seven studies, a large degree of LVEF improvement was observed in the image-guided group (WMD, 3.25 [95% CI, 1.80 to 4.70]; *p* < 0.01, Fig. 3) with low heterogeneity (P = 0.15; I^2^ = 37%), when compared with the routine CRT implantation group.

#### Reduction in LVESV

**Fig. 4:**
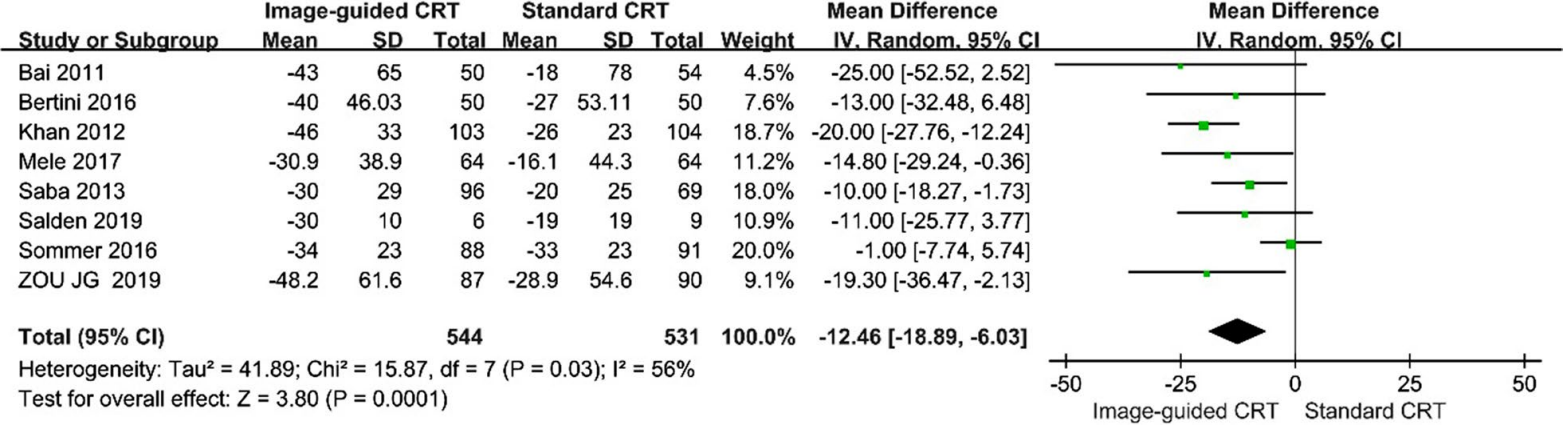
Forest plot of change in LVESV between groups. A random-effects model and inverse variance (IV) method were used to pool data. Abbreviations as in Fig. 3

Eight studies compared the changes from baseline to post-treatment LVESV in the image-guided group with the changes in the standard group [15–22]. Six studies [15–18, 20, 21] demonstrated that the decrease of LVESV was more prominent in the image-guided group. Pooled data illustrated the comparative results from this inter-group meta-analysis (WMD, − 12.46[95% CI, − 18.89 to − 6.03]; *p* < 0.01, Fig. 4). The homogeneity testing showed moderate differences between trials (P = 0.03; I^2^ = 56%).

#### HF hospitalization and mortality rate

**Fig. 5:**
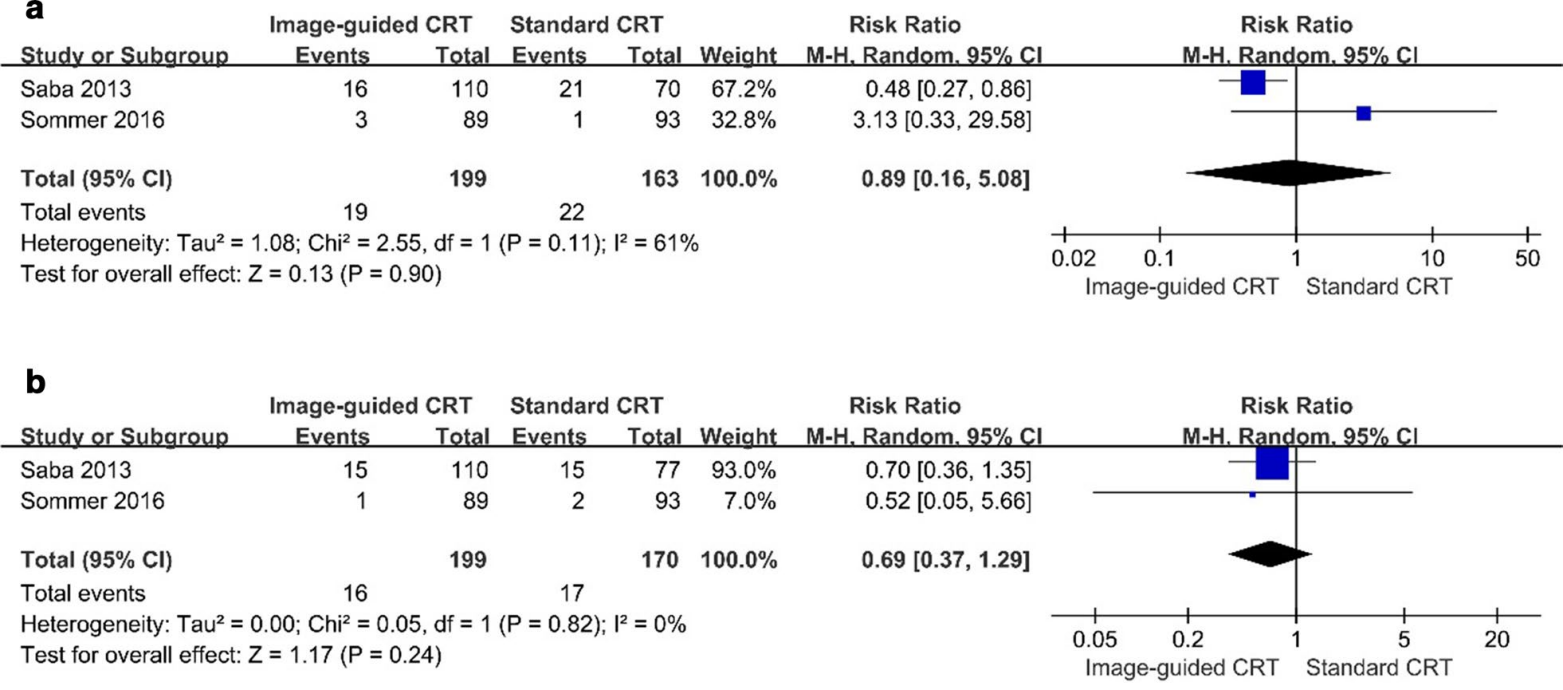
**a** Forest plot of **a** HF hospitalization between groups, **b** mortality rate between groups. A random-effects model and Mantel–Haenszel method were used to pool data. Abbreviations as in Fig. 2

Two studies directly compared HF hospitalization and mortality rates between the image-guided group and the standard group [17, 19]. Pooled data demonstrated no difference when both outcome measures were compared respectively between the two groups (HF Hospitalization: RR, 0.89 [95% CI, 0.16 to 5.08]; *p* = 0.90, Fig. 5a. Mortality rate: RR, 0.69 [95% CI, 0.37 to 1.29]; *p* = 0.24, Fig. 5b).

#### Concordance of LV

**Fig. 6:**
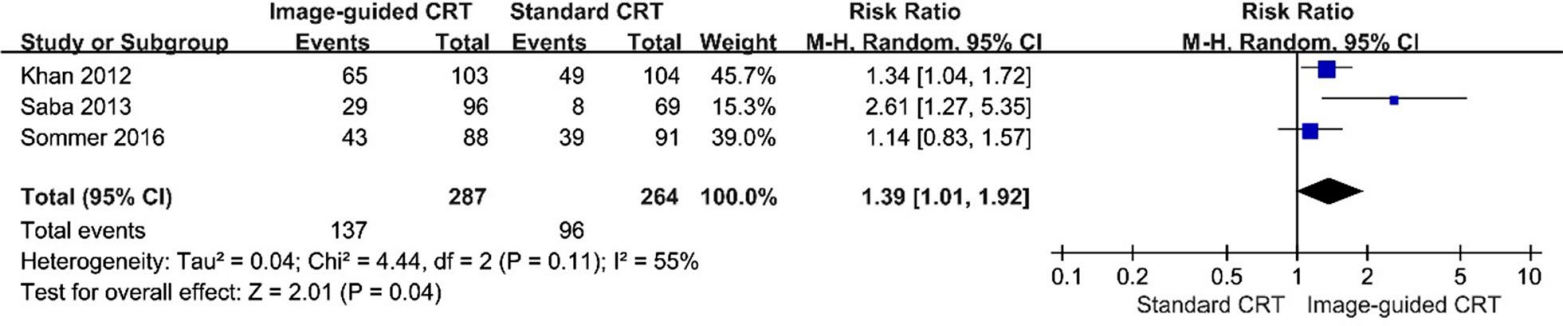
Forest plot of concordance of LV between groups. A random-effects model and Mantel–Haenszel method were used to pool data. Abbreviations as in Fig. 2

Three studies compared the concordance of LV in the image-guided group with that in the standard group [16, 17, 19]. Pooled data demonstrated significant difference in this parameter when the image-guided treatment group were compared with the control group (RR, 1.39 [95% CI, 1.01 to 1.92]; *p* < 0.05, Fig. 6). Meanwhile, there was medium heterogeneity (P = 0.11; I^2^ = 55%).

### Subgroup analysis

Subgroup analysis was performed across several different variables to determine the origin of this heterogeneity. The differences in CRT responses (Table 4) and the LVEF changes (Additional file 2: Table S2) between the image-guided group and the standard group demonstrated that statistically significant associations exist in all subgroups. In contrast, the level was affected by study design, country, baseline LVEF, and baseline LVESV. The reduction of LVESV (Additional file 3: Table S3) between groups showed a statistically significant association based on country and study quality.

**Table 4:** Subgroup analysis for the association of CRT response between groups for each variable

Variable	Subgroups	No. of studies	No. of patients CRT response (Total)	Test of relationship		Heterogeneity (%)	*p* value for heterogeneity	*p* value between subgroups
				RR (95%CI)	*p* value			
Country	United states or Europe	6	542 (883)	1.37 (1.23–1.52)	< 0.01	0	0.76	0.24
	Asia	1	123 (177)	1.20 (0.98–1.46)	< 0.01	–	–	
Study design	RCT	4	444 (728)	1.30 (1.15–1.46)	< 0.01	0	0.57	0.39
	observational	3	221 (332)	1.41 (1.21–1.65)	< 0.01	0	0.66	
LVEF (%)	≥ 25	4	341 (570)	1.39 (1.21–1.60)	< 0.01	12	0.33	0.37
	< 25	3	324 (490)	1.33 (1.21–1.45)	< 0.01	0	0.98	
LVESV (ml)	≥ 150	4	447 (667)	1.25 (1.13–1.40)	< 0.01	0	0.95	0.08
	< 150	3	218 (393)	1.52 (1.26–1.84)	< 0.01	0	0.78	
Techniques	ECHO	3	389 (584)	1.32 (1.17–1.48)	< 0.01	0	0.5	0.73
	Non-ECHO	4	276 (476)	1.36 (1.17–1.59)	< 0.01	0	0.52	

RR, risk ratio; ECHO, Echocardiography; other abbreviations as in Table. 2

#### Publication bias

**Fig. 7:**
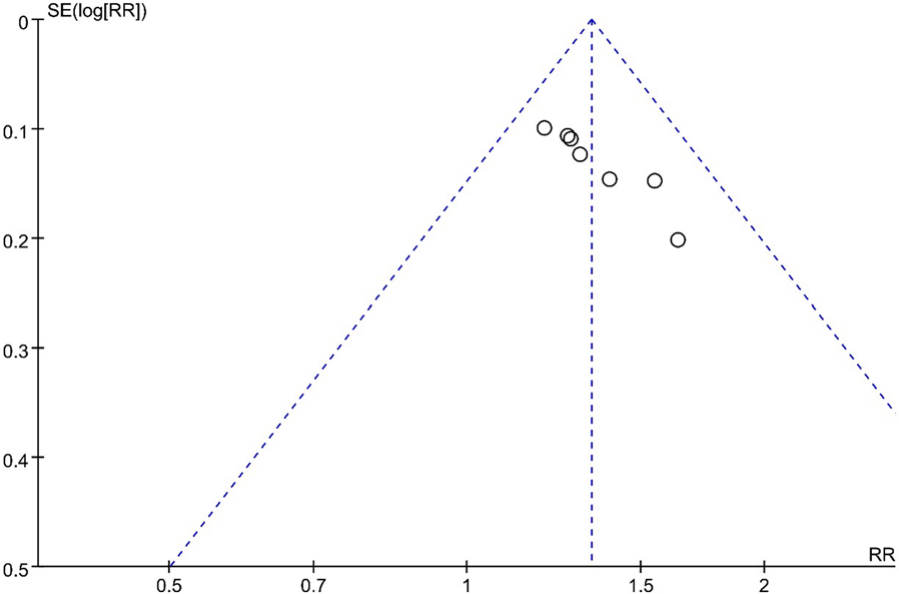
Funnel plot of CRT response between groups

Funnel plots did not suggest publication bias for any of the outcomes (CRT response, Fig. 7; Reduction in LVESV, Additional file 4: Figure S1, and Improvement in LVEF, Additional file 5: Figure S2).

### Sensitivity analysis

Sensitivity analysis indicated that none of the exclusions of a specific study would change the direction or magnitude of the summary effect for the correlation of image-guided CRT treatment with CRT responses, as well as changes in LVESV and LVEF. Sommer et al. [19] showed that the changes in LVESV and LVEF between groups did not show a significant difference. Exclusion of this trial resulted in the heterogeneity of 0%. The heterogeneity of concordance of LV was inconsistent after sequentially excluding each study. Removal of study by Saba et al. [17] contributed to more homogeneous results (P = 0.44; I^2^ = 0).

## Discussion

This study provides a meta-analysis of published literature in which imaging guidance was applied in CRT placement. Our findings illustrated that this technique is associated with an increase in CRT efficacy among HF patients. It is also associated with a more preferable concordance of LV, a greater reduction of LVESV, as well as a higher increase in LVEF, when compared to the standard CRT placement group. No differences in HF hospitalization and mortality rates were identified between both groups.

Our results were in agreement with a previously published meta-analysis on this topic [23]. It is worth noting that this previous meta-analysis included a much smaller subject pool totaling 500 patients to explore the effect of imaging techniques on the efficacy of CRT. Our study has a larger sample size. We also looked specifically at subgroups of HF patients and evaluated the concordance of LV between groups. Pooling showed that the image-guided group had a significantly higher concordance of LV than the standard group. Especially in the guided group, CRT significantly reduced LVESV and increased LVEF. Only one trial by Sommer et al. [19] could not show a favorable effect on reversing LV remodeling in the image-guided group. The discrepancy may be explained by differences in the study design and patient selection. Pooling could also benefit from the large sample size to decide whether imaging techniques has a significant association with this outcome.

Patient with appropriate indications for CRT remains a vital factor for achieving greater therapeutic responses. Maass et al. [24] applied the CAVIAR response score to predict the amount of reverse remodeling after CRT. Lower age, larger QRS area, longer interventricular mechanical delay, and presence of apical rocking were identified as independent predictors of response; all represented in the CAVIAR response score. This score may be used to improve patient selection and predict the clinical outcome before CRT implantation. Despite a volumetric improvement, we found that the imaging group showed no pronounced differences in major clinical outcomes such as hospitalization and mortality rates, when compared to the standard CRT group. This may be explained by the lack of contemporary data in HF hospitalization and mortality rates from limited number of clinical trials to date. Reverse ventricular remodeling, on the other hand, is commonly associated with clinical endpoints such as heart failure hospitalizations and all-cause mortality. Foley PW et al. [25] demonstrated that LV reverse remodeling was an independent predictor of morbidity and mortality for up to 5 years after CRT implantation and the authors demonstrated that pump failure was mainly responsible for this association. The CAVIAR response score were also shown to predict clinical events including all-cause mortality and HF hospitalizations [24]. It predicted the incidence with < 2% adverse clinical events with a CAVIAR score > 4 and more than 20% events if CAVIAR is < 2 in super-responders in the first year. It suggested that guiding LV lead placement to the latest activation site was not the only factor associated with clinical outcomes. Additional factors including non-ischemic cardiomyopathy, myocardial scar distribution, QRS duration, and LBBB QRS morphology may have added influences on the results [26].

In addition, concordance of LV could only be achieved in part of the patient in the image-guided group. According to the STARTER trial [17], only 30% of the recommended segments were consistent with the location of the LV lead. A considerable percentage of the image-guided group even made the LV lead placed in the scar. This LV lead position distinction was linked with poor clinical outcomes and may explain the disagreement in volumetric responses after CRT. The optimal placement of an LV lead into the coronary sinus (CS) may be impossible in some cases, and left phrenic nerve (LPN) stimulation may occur in a specific position. Due to these challenges, transseptal endocardial and surgical epicardial lead placement may become alternatives to conventional LV lead implantation [27]. Transseptal endocardial LV lead placement does bring some advantages: transvenous access, endocardial pacing, more lead placement sites, and there is less concern for compromising LPN stimulation or LV pacing threshold for positional stability. The surgical epicardial lead placement is typically used by either mini-thoracotomy or video-assisted thoracoscopy. In the future, large randomized trials are required to assess the prospective benefits of alternative LV pacing techniques to improve the rate of optimal lead positions.

Evaluating LV mechanical and electrical dyssynchrony is essential to determine CRT response [28]. The transmission of electrical activity is parallel to the mechanical activation, so the duration and morphology of QRS can reveal the dyssynchronization of LV electrical and mechanical activities. It can be used as an electrocardiographic indicator to predict the CRT response [29]. The level of LV electric delay is evaluated based on the interval of the surface (lead II) of the QRS at the first main peak (positive or negative) of LV, which is associated with reduced mitral regurgitation, leading to the development of a leading strategy for the LV to improve CRT response [30]. As compared to other sites on the myocardial wall, pacing in the most delayed mechanical activated site can shorten the total electromechanical activation time of LV [16]. Another study showed that the location of the latest activated regions varies, with 67% of patients located in the posterolateral myocardial wall and the remaining 33% in different regions [31].

It is a big challenge to place the LV lead in the targeted position through the coronary vein [16]. Imaging techniques have been assisted in determining this location, such as CMR imaging [11], ECHO [9, 10], and nuclear imaging [7, 32]. The ECHO can be intuitive to evaluate left ventricular synchrony, thus predict the efficacy of CRT treatment for heart failure patients accurately. Several clinical trials have incorporated ECHO and fluoroscopic venography to guide LV placement. In the TARGET trial [16], fluoroscopic venograms with a steep left anterior oblique (LAO) were aligned with the short axis parastnum ECHO using a two-dimensional visual correspondence approach in the guided group. The anatomy of the CS similar to the short-axis of ECHO was displayed by the LAO fluoroscopic venography image, which assisted operators to match the suitable vein with the ideal segment under the guidance of the ECHO. Consequently: 64%, 26%, and 10% of patients placed the LV lead in the recommended, suboptimal, and inappropriate locations, respectively. However, this technique relies heavily on the operator experience and has poor repeatability.

Programmed with stimulated echoes, CMR can deliver high-quality circumferential strain data to define the status of mechanical dyssynchrony. In combination with scar evaluation by late gadolinium enhancement, CMR can advance current criteria to determine optimal LV lead placement [33]. Salden et al. [20] assessed Real-time image-guided LV lead placement by fusion of fluoroscopy images with CMR images during CRT. Real-time visualization of the latest contracting area, scar location, and LPN position were identified on a custom-made treatment-guidance platform (CARTBox, CART-Tech B.V., Utrecht, The Netherlands) from pre-procedurally acquired CMR and computed tomography (CT) scans. Based on the delayed activation, location of the scar, and the LPN, a target area for LV lead implantation was chosen. After 3D image fusion of the 3D-treatment dataset with fluoroscopy, the LV lead targets and scar segments together with LPN and coronary ostium are visualized on live fluoroscopy during the LV lead implantation. Thus it could assist the cardiologist in achieving image-guided LV lead placement in a targeted area. However, CMR is not suitable for patients with a pacemaker, and the inspection time is relatively long.

Several studies have confirmed that SPECT can be used to better evaluate left ventricular dyssynchrony in recent years [34, 35], and it is much more reproducible than echocardiography. SPECT can measure mechanical dyssynchrony, myocardial activity, and LV function in one scan. Thus, SPECT MPI and positron emission tomography (PET) are regarded as the "one-stop-shop" for CRT guidance [7, 32, 36]. The GUIDECRT trial by Zou et al. [21] also validated the improvement of CRT efficacy guided by SPECT. Whether the exact LV lead concordance using image-guided techniques would lead to improved clinical outcomes after CRT remains to be confirmed. Merging of target segments by image-guided techniques with electrophysiological mapping may bring more promising outcomes in the future.

Several limitations of this study should be considered. Firstly, observational studies carry an inherent bias against the incidence of CRT response. Secondly, the definitions of responses in the enrolled studies are inconsistent among analyzed studies, which may further impact the extents of reported changes in LVESV and LVEF between groups. Lastly, although several CRT trials have yielded promising results, more randomized, prospective multicenter trials are needed to validate these new techniques before their widespread applications in standardized clinical practices.

## Conclusions

This meta-analysis indicates that image-guided CRT is correlated with improved CRT volumetric response and cardiac function in heart failure patients but not with lower hospitalization or mortality rate. Further large randomized prospective clinical trials are required to prove a causal relationship between this innovative technique and overall clinical benefits.

## Supplementary Information

**Additional file 1: Table S1.** PRSIMA checklist.

**Additional file 2: Table S2.** Subgroup analysis for the association of changes in LVEF between groups for each variable.

**Additional file 3: Table S3.** Subgroup analysis for the association of changes in LVESV between groups for each variable.

**Additional file 4: Figure S1.** Funnel plot of reduction in LVESV between groups.

**Additional file 5: Figure S2.** Funnel plot of improvement in LVEF between groups.

## Data Availability

The datasets generated and analyzed during the current study are available from the corresponding author on reasonable request.

## Abbreviations

CRT: Cardiac resynchronization therapy
CRT-P: CRT pacemaker
CRT-D: CRT defibrillator
LVEF: Left ventricular ejection fraction
LVESV: Left ventricular end-systolic volume
LV: Left ventricular
HF: Heart failure
RCTs: Randomized controlled trials
CI: Confidence interval
RR: Risk ratio
SD: Standard deviation
WMD: Weighted mean difference
CMR: Cardiac magnetic resonance
ECHO: Echocardiography
STE: Speckle tracking ECHO
PA: Phase analysis
SPECT MPI: Single-photon emission computed tomography myocardial perfusion imaging
VVI: Vector velocity imaging
NYHA: New York Heart Association
Concordance of LV: Concordance of LV lead to the latest sites of contraction
LPN: Left phrenic nerve
LAO: Left anterior oblique
