# Supplementary material for: Comparative efficacy of image-guided techniques in cardiac resynchronization therapy: a meta-analysis
Source: BMC Cardiovasc Disord. 2021 May 24;21:255. doi: 10.1186/s12872-021-02061-y (PMC8142495; doi:10.1186/s12872-021-02061-y)
Supplement: Supplementary file 2 — Additional file 2: Table S2. Subgroup analysis for the association of changes in LVEF between groups for each variable. [file 12872_2021_2061_MOESM2_ESM.docx]

Additional file 2: **Table S2** Subgroup analysis for the association of changes in LVEF between groups for each variable

| Variable | Subgroups | | No. of  studies | Test of relationship  WMD (95%CI) P value | Heterogeneity (%) | P value for  heterogeneity | P value between subgroups |
| --- | --- | --- | --- | --- | --- | --- | --- |
| Study design | | RCT  observational | 3  4 | 2.03(0.61 to 3.45) <0.01  4.73(3.01 to 6.45) <0.01 | 47  0 | 0.15  0.99 | 0.02 |
| LVEF (%) | | ≥25  <25 | 4  3 | 4.16(2.44 to 5.88) <0.01  2.41(0.99 to 3.83) <0.01 | 0  68 | 0.86  0.15 | 0.12 |
| LVESV (ml) | | ≥150  <150 | 3  4 | 2.41(0.99 to 3.83) <0.01  4.16(2.44 to 5.88) <0.01 | 68  0 | 0.15  0.86 | 0.12 |

Abbreviations: WMD: Weighted mean difference; other abbreviations as in Table. 2
