# Supplementary material for: Comparative efficacy of image-guided techniques in cardiac resynchronization therapy: a meta-analysis
Source: BMC Cardiovasc Disord. 2021 May 24;21:255. doi: 10.1186/s12872-021-02061-y (PMC8142495; doi:10.1186/s12872-021-02061-y)
Supplement: Supplementary file 3 — Additional file 3: Table S3. Subgroup analysis for the association of changes in LVESV between groups for each variable. [file 12872_2021_2061_MOESM3_ESM.docx]

Additional file 3: **Table S3** Subgroup analysis for the association of changes in LVESV between groups for each variable

| Variable | Subgroups | No. of  studies | Test of relationship | | Heterogeneity (%) | P value for  heterogeneity | P value between subgroups |
| --- | --- | --- | --- | --- | --- | --- | --- |
|  |  |  | WMD (95%CI) | P value |  |  |  |
| Country | United states or Europe  Asia | 7  1 | -11.82(-18.72 to -4.93) <0.01  -19.30(-36.47 to -2.13) 0.03 | | 60  - | 0.02  - | 0.43 |
| Study design | RCT  observational | 4  4 | -11.70(-21.54 to -1.87) 0.02  -14.15(-22.81 to -5.49) <0.01 | | 79  0 | <0.01  0.85 | 0.71 |
| LVEF (%) | ≥25  <25 | 5  3 | -12.23(-18.01 to -6.46) <0.01  -13.21(-29.34 to 2.93) 0.11 | | 0  86 | 0.90  <0.01 | 0.91 |
| LVESV (ml) | ≥150  <150 | 4  4 | -14.38(-27.55 to -1.21) 0.03  -11.33(-17.46 to -5.20) <0.01 | | 81  0 | <0.01  0.95 | 0.68 |

Abbreviations: WMD: Weighted mean difference; other abbreviations as in Table. 2
