# Supplementary figures and images for: Comparative efficacy of image-guided techniques in cardiac resynchronization therapy: a meta-analysis
Source: BMC Cardiovasc Disord. 2021 May 24;21:255. doi: 10.1186/s12872-021-02061-y (PMC8142495; doi:10.1186/s12872-021-02061-y)

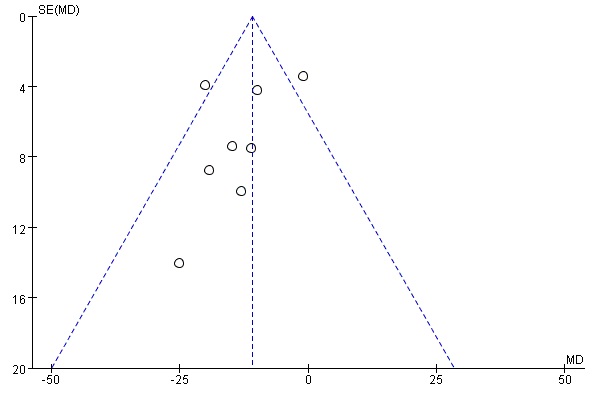


Additional file 4: **Fig. S1** Funnel plot of reduction in LVESV between groups

Supplement: Supplementary file 4 — Additional file 4: Figure S1. Funnel plot of reduction in LVESV between groups. [file 12872_2021_2061_MOESM4_ESM.docx]

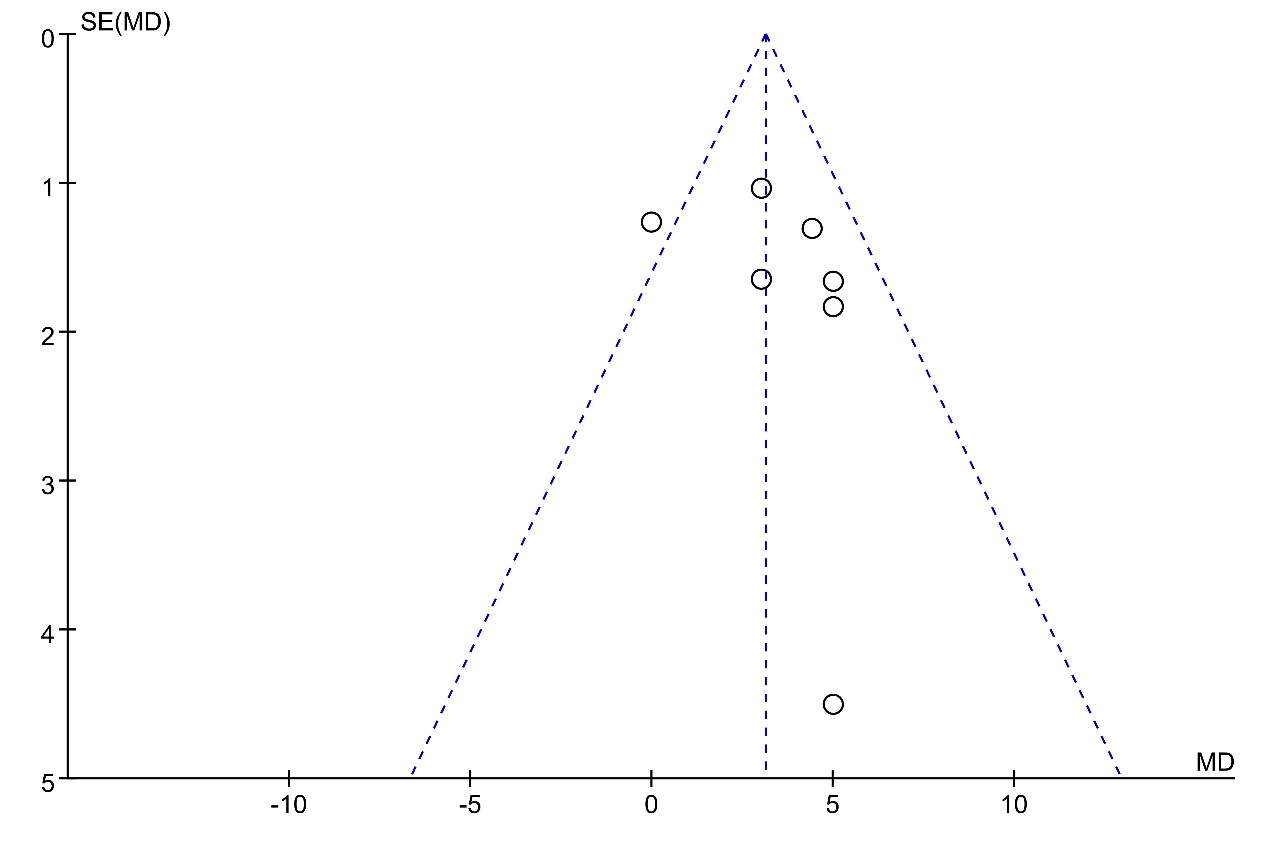


Additional file 5: **Fig. S2** Funnel plot of improvement in LVEF between groups

Supplement: Supplementary file 5 — Additional file 5: Figure S2. Funnel plot of improvement in LVEF between groups. [file 12872_2021_2061_MOESM5_ESM.docx]
